# Elevated expression of Cripto-1 correlates with poor prognosis in hepatocellular carcinoma

**DOI:** 10.18632/oncotarget.5057

**Published:** 2015-09-05

**Authors:** Jia-hong Wang, Wei Wei, Jing Xu, Zhi-xing Guo, Cheng-zuo Xiao, Yong-fa Zhang, Pei-en Jian, Xiao-liang Wu, Ming Shi, Rong-ping Guo

**Affiliations:** ^1^ Department of Hepatobilliary Oncology, Sun Yat-sen University Cancer Center, State Key Laboratory of Oncology in South China, Collaborative Innovation Center for Cancer Medicine, Guangzhou, China; ^2^ State Key Laboratory of Oncology in South China, Sun Yat-sen University Cancer Center, Collaborative Innovation Center for Cancer Medicine, Guangzhou, China; ^3^ Department of Ultrasonics, Sun Yat-sen University Cancer Center, State Key Laboratory of Oncology in South China, Collaborative Innovation Center for Cancer Medicine, Guangzhou, China

**Keywords:** hepatocellular carcinoma, Cripto-1, MMP-9, aggressiveness, prognosis

## Abstract

Cripto-1 could promote tumorigenesis in a wide range of carcinomas, yet little is known in hepatocellular carcinoma (HCC). The expression of Cripto-1 and MMP-9 were assessed by immunohistochemistry in 205 HCC specimens. The correlation between Cripto-1 and MMP-9, clinicopathological/prognostic value in HCC was examined. Cripto-1 overexpression was correlated with larger tumor, TNM stage, BCLC stage and tumor recurrence. In multivariate analyses, Cripto-1 was an independent predictor for overall survival (OS) and time to recurrence (TTR). Cripto-1 expression was increased in TNM and BCLC stage-dependent manner. Cripto-1 overexpression was associated with poor prognosis in patients subgroups stratified by tumor size, tumor differentiation, TNM and BCLC stage. In addition, Cripto-1 was positively correlated with MMP-9 among 205 HCC samples. Patients with Cripto-1 upregulation had poor OS and shorter TTR in low and high aggressiveness groups. Furthermore, Cripto-1 had predictive validity for early and late recurrence in HCC patients. Combination of Cripto-1 and serum AFP was correlated with OS and TTR. In conclusion, Cripto-1 overexpression contributes to aggressiveness and poor prognosis of HCC. Cripto-1/AFP expression could be a potential prognostic biomarker for survival in HCC patients.

## INTRODUCTION

Hepatocellular carcinoma (HCC) is one of the most common highly invasive malignant tumors associated with high recurrence incidence and poor prognosis [1, 2]. Hepatic resection is a main modality for curative therapy, however, the prognosis of HCC patients after surgical resection remains unsatisfactory, with a 5-year recurrence rate ranged from 65% to 80% [3–5]. Prediction of recurrence remains a great challenge for HCC patients after hepatectomy. Although some clinicopathologic features of HCC, such as vascular invasion and tumor multifocality, could be used to assess the prognosis of HCC patients, they cannot meet clinical requirements for precise prediction of HCC course [6]. Indeed, survival may vary widely among HCC patients with the same clinicopathologic characteristics. Therefore, in an attempt to predict outcomes of HCC patients, there is an urgent need for identifying molecular markers of HCC development and progression.

Cripto-1, also known as teratocarcinoma-derived growth factor-1 (TDGF-1) [7], is a glycosylphosphatidylinositol (GPI)-anchored membrane protein [8, 9], which acts as a coreceptor for the transforming growth factor-β (TGF-β) subfamily of ligands Nodal, growth differentiation factor-1 and -3 (GDF1 and GDF3). Cripto-1 could promote the development of vertebrate and the progression of malignant tumors [10–12]. Recently, it has been reported that plasma Cripto-1 might represent a novel biomarker for the detection of breast and colon carcinomas [13]. Cripto-1 overexpression is correlated with poor prognosis in gastric cancer [14]. However, the role of Cripto-1 in prognosis of HCC patients has not been well clarified.

In this study, we demonstrated the expression of Cripto-1 in HCC tissues by immunohistochemistry (IHC). Correlation of Cripto-1 with clinicopathological parameters and prognosis of HCC patients were analysed. In addition, it has been known that MMP-9 is closely participated in capsular infiltration and metastasis in HCC [15] and serum AFP level is an unfavorable prognostic factor for HCC patients [16]. Therefore, we also estimated the association of Cripto-1 with MMP-9 protein and investigated the prognostic value of Cripto-1 combined with serum AFP level in HCC patients.

## RESULTS

### Cripto-1 expression in HCC

**Figure 1:**
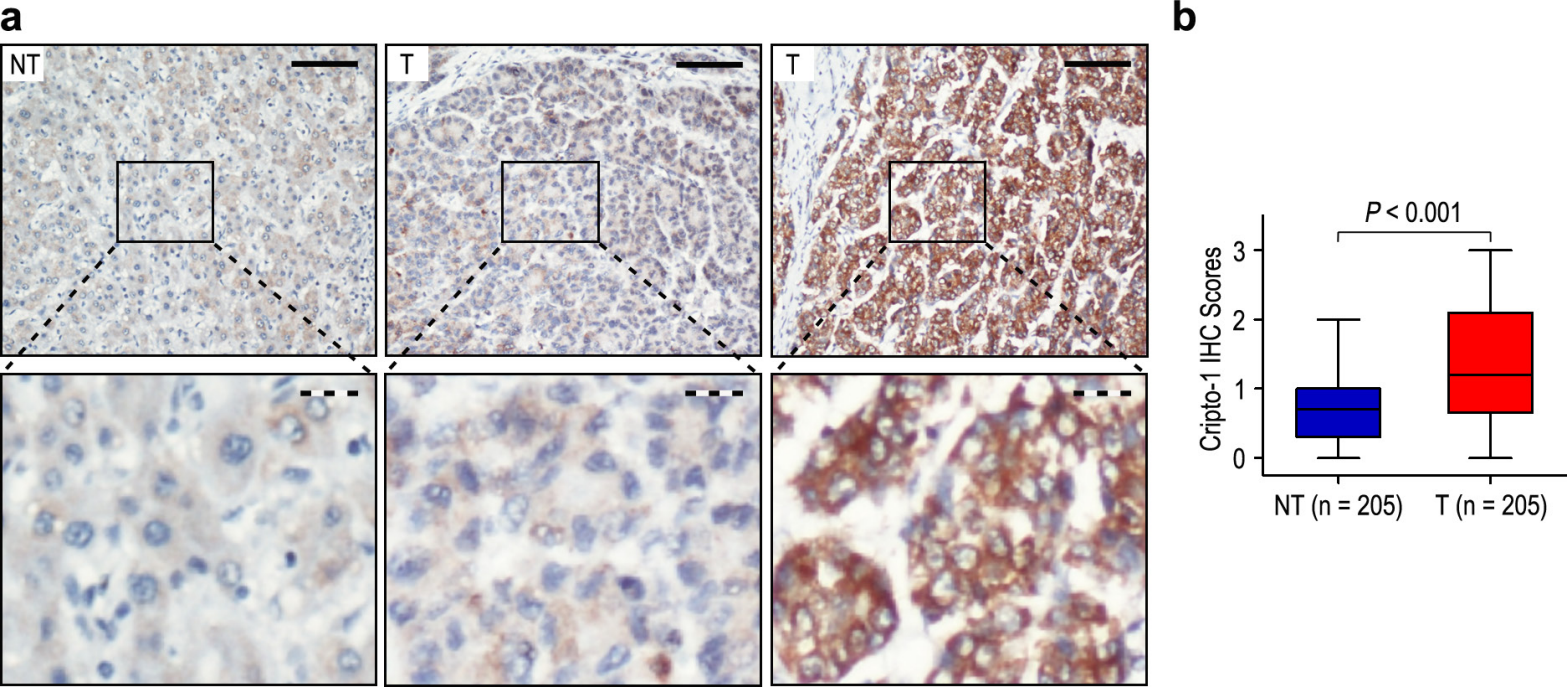
Cripto-1 was significantly up-regulated in hepatocellular carcinoma (HCC) **a.** Immunohistochemistry (IHC) assays of Cripto-1 expression in 205 paired HCC and adjacent non-tumorous tissues. The upper left panel represents low Cripto-1 expression in adjacent non-tumorous tissues. The upper middle and right panel represents low and high Cripto-1 expression in HCC. Lower panels represent magnified pictures of boxed area in the corresponding upper panels. The full line and dotted line scale bar represents 50 μm and 10 μm, respective. **b.** Cripto-1 expression levels were compared with HCC and adjacent non-tumorous specimens. Statistical analysis was performed by Paired-Samples *t*-test.

To elucidate the biological significance of Cripto-1 in HCC, we examined the immunohistochemical expression of Cripto-1 in 205 HCC specimens, as compared with the levels in matched adjacent non-tumorous liver tissues. The results showed that Cripto-1 was primarily localized in the cytoplasm of tumor cells (Figure 1a). High Cripto-1 expression was found in 102 of the 205 (49.8%) primary HCC specimens, compared with 26/205 (12.7%) in adjacent non-tumorous tissues (*P* < 0.001; Figure 1b). In addition, we also compared staining of non-tumor and tumor tissues in Cripto-1 highly positive cases and found that the expression of Cripto-1 in tumor tissues was higher than that in non-tumor tissues. (Data not show) These data suggested that Cripto-1 expression was significantly higher in HCC tissues than that in adjacent non-tumorous tissues.

### Correlation of Cripto-1 with clinicopathological variables

To verify the functions of Cripto-1 in HCC, we correlated Cripto-1 status in 205 HCC samples with widely recognized clinicopathological features. The high expression of Cripto-1 in HCC was associated with larger tumor (>5 cm in diameter) (*P* < 0.001), TNM stage (*P* = 0.018), BCLC stage (*P* < 0.001) and tumor recurrence (*P* < 0.001) (Table 1). In contrast, Cripto-1 expression displayed no association with gender, age, AFP, HBsAg, gamma-glutamyltransferase (GGT), liver cirrhosis, tumor number, satellite nodule, tumor capsule, tumor differentiation and vascular invasion (all *P* > 0.05).

**Table 1:** Correlation of Cripto-1 protein expression with clinicopathological parameters

Characteristics	No. of patients	Cripto-1 expression (%)		*P*-value
		Low	High	
**Gender**				
Female	24	11 (45.8%)	13 (54.2%)	0.646
Male	181	92 (50.8%)	89 (49.2%)	
**Age (years)**				
≤50	109	50 (45.9%)	59 (54.1%)	0.182
>50	96	53 (55.2%)	43 (44.8%)	
**AFP (ng/ml)**				
≤400	119	64 (53.8%)	55 (46.2%)	0.233
>400	86	39 (45.3%)	47 (54.7%)	
**HBsAg**				
Negative	13	6 (46.2%)	7 (53.8%)	0.761
Positive	192	97 (50.5%)	95 (49.5%)	
**GGT (U/l)**				
≤50	109	61 (56.0%)	48 (44.0%)	0.081
>50	96	42 (43.8%)	54 (56.2%)	
**Liver cirrhosis**				
No	45	27 (60.0%)	18 (40.0%)	0.138
Yes	160	76 (47.5%)	84 (52.5%)	
**Tumor size (cm)**				
≤5	119	74 (62.2%)	45 (37.8%)	<0.001
>5	86	29 (33.7%)	57 (66.3%)	
Tumor number*				
Single	189	97 (51.3%)	92 (48.7%)	0.288
Multiple	16	6 (37.5%)	10 (62.5%)	
**Satellite nodule**				
No	178	94 (52.8%)	84 (47.2%)	0.059
Yes	27	9 (33.3%)	18 (66.7%)	
**Tumor capsule**				
No/incomplete	121	59 (48.8%)	62 (51.2%)	0.610
Complete	84	44 (52.4%)	40 (47.6%)	
**Tumor differentiation**				
I–II	142	71 (50.0%)	71 (50.0%)	0.916
III–IV	63	32 (50.8%)	31 (49.2%)	
**Vascular invasion**				
No	185	97 (52.4%)	88 (47.6%)	0.057
Yes	20	6 (30.0%)	14 (70.0%)	
**TNM stage**				
I	160	87 (54.4%)	73 (45.6%)	0.018
II	10	6 (60.0%)	4 (40.0%)	
III	35	10 (28.6%)	25 (71.4%)	
**BCLC stage**				
0	18	11 (61.1%)	7 (38.9%)	<0.001
A	95	61 (64.2%)	34 (35.8%)	
B	71	26 (36.6%)	45 (63.4%)	
C	21	5 (23.8%)	16 (76.2%)	
**Recurrence status**				
No	93	67 (72.0%)	26 (28.0%)	<0.001
Early recurrence	78	19 (24.4%)	59 (75.6%)	
Late recurrence	34	17 (50.0%)	17 (50.0%)	
**MMP-9 expression**				
Low	109	79 (72.5%)	30 (27.5%)	<0.001
High	96	24 (25.0%)	72 (75.0%)	

* Tumor number indicates number of primary tumor mass detected at the time of surgical operation.

### Overexpression of Cripto-1 significantly associated with poor prognosis in HCC patients

Furthermore, to confirm the effect of Cripto-1 status on OS and TTR in HCC patients, we analyzed the correlation between traditional clinicopathologic parameters and patients outcomes by univariate analysis. The results showed that high expression of Cripto-1 (*P* < 0.001), high AFP level (*P* < 0.001), high GGT level (*P* = 0.009), liver cirrhosis (*P* = 0.006), larger tumor size (*P* < 0.001) and vascular invasion (*P* < 0.001) were unfavourable predictors for OS of HCC patients. In addition, Kaplan-Meier analysis demonstrated that high Cripto-1 expression (*P* < 0.001), high AFP level (*P* = 0.001), high GGT level (*P* = 0.016), liver cirrhosis (*P* = 0.012), larger tumor size (*P* < 0.001), satellite nodule (*P* = 0.007) and vascular invasion (*P* < 0.001) were significantly associated with shorter TTR in HCC patients (Table 2).

**Table 2:** Univariate and multivariate analysis of Cripto-1 associated with survival and recurrence in HCC patients

Variables*	OS				TTR			
	Univariate	Multivariate			Univariate	Multivariate		
	*P*-value	*P*-value	HR	95% CI	*P*-value	*P*-value	HR	95% CI
Gender (Female vs. Male)	NS	NS			NS	NS		
Age, years (≤50 vs. >50)	NS	NS			NS	NS		
AFP (ng/mL) (≤400 vs. >400)	<0.001	<0.001	2.187	1.447–3.305	0.001	0.001	1.876	1.279–2.750
HBsAg (Negative vs. Positive)	NS	NS			NS	NS		
GGT (U/l) (≤50 vs. >50)	0.009	NS			0.016	NS		
Liver cirrhosis (No vs. Yes)	0.006	0.013	2.182	1.176–4.049	0.012	NS		
Tumor size (cm) (≤5 vs. >5)	< 0.001	NS			<0.001	0.050	1.559	0.999–2.433
Tumor number (Single vs. Multiple)	NS	NS			NS	NS		
Satellite nodule (No vs. Yes)	NS	NS			0.007	NS		
Tumor capsule (No/ incomplete vs. Complete)	NS	NS			NS	NS		
Tumor differentiation (I–II vs. III–IV)	NS	NS			NS	NS		
Vascular invasion (No vs. Yes)	<0.001	<0.001	5.031	2.788–9.077	<0.001	<0.001	4.951	2.790–8.785
Cripto-1 (Low *versus* High)	<0.001	0.001	2.198	1.408–3.431	<0.001	<0.001	3.036	1.998–4.616

* TNM stage and BCLC stage was combined with several clinical indexes such as tumor size, number and tumor thrombus; we did not enter the TNM stage and BCLC stage into multiple analysis with these indexes to avoid any bias in analysis.

GGT gamma-glutamyltransferase, AFP α-fetoprotein, OS overall survival, TTR time to recurrence, NS not significant, HR hazard ratio, CI confidential interval.

**Figure 2:**
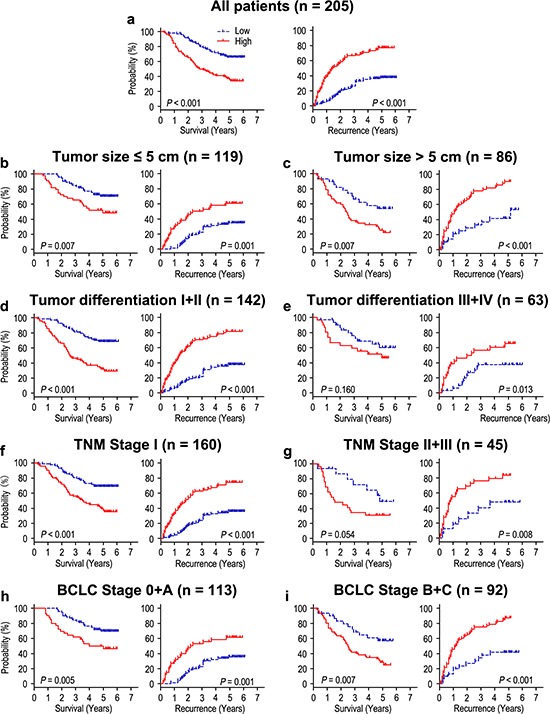
Overall survival and time to recurrence are shown for patients with HCC All patients were stratified according to tumor size, tumor differentiation, TNM classification and BCLC stage. Kaplan-Meier survival estimates and log-rank tests were used to analyze the prognostic significance of Cripto-1 expression in all patients **a.** and each subgroup **b–i.**

Significant OS and TTR advantages were observed for the HCC patients with low Cripto-1 expression (both *P* < 0.001) (Figure 2a). In addition, the median of OS times in Cripto-1 high-level group (*n* = 102) and Cripto-1 low-level group (*n* = 103) were 32.5 months and 57.0 months, while the median of the TTR were 16.5 months and 56.0 months. Furthermore, the 5-year OS and TTR rates of the Cripto-1 high-level group were 37.1% and 23.0%, which were significantly lower than those of the Cripto-1 low-level group (65.9% and 62.3%) (Figure 2a). Moreover, the expression levels of Cripto-1 in tumors increased in TNM and BCLC stage-dependent manner, and they were significantly higher in TNM stage III and BCLC stage C tumors than those in TNM stage I and BCLC stage 0 tumors (Supplementary Figure S1).

In addition, we analysed whether Cripto-1 could be an independent predictors for OS and TTR in HCC patients. A multivariate Cox model adjusted for AFP, GGT, liver cirrhosis, tumor size, satellite nodule, vascular invasion and Cripto-1 expression was performed. Our results revealed that overexpression of Cripto-1 was a poor independent predictor for OS in HCC patients (HR = 2.198, 95% CI = 1.408–3.431, *P* < 0.001). Moreover, the patients with high Cripto-1 expression was almost 3.0 times more likely to suffer from relapse than those with low Cripto-1 expression (HR = 3.036, 95% CI = 1.998–4.616, *P* < 0.001) (Table 2).

To further investigate the prognostic value of Cripto-1 in different subgroups, patients were stratified according to tumor size (Figure 2b, 2c), tumor differentiation (Figure 2d, 2e), TNM stage (Figure 2f, 2g) and BCLC stage (Figure 2h, 2i). The overexpression of Cripto-1 maintained its prognostic value in predicting shorter OS and TTR in all of these subgroups for except OS in patients who had tumor differentiation III/IV (*P* = 0.160) or TNM stage II/III (*P* = 0.054). Therefore, it suggests that Cripto-1 may serve as a potential prognostic biomarker for HCC patients in different risk groups.

### Overexpression of Cripto-1 predicts poor prognosis independent of tumor aggressiveness

To better understand the clinical significance of Cripto-1 on progression in HCC, we evaluated the correlation of Cripto-1 and MMP-9 expression in HCC patients.

**Figure 3:**
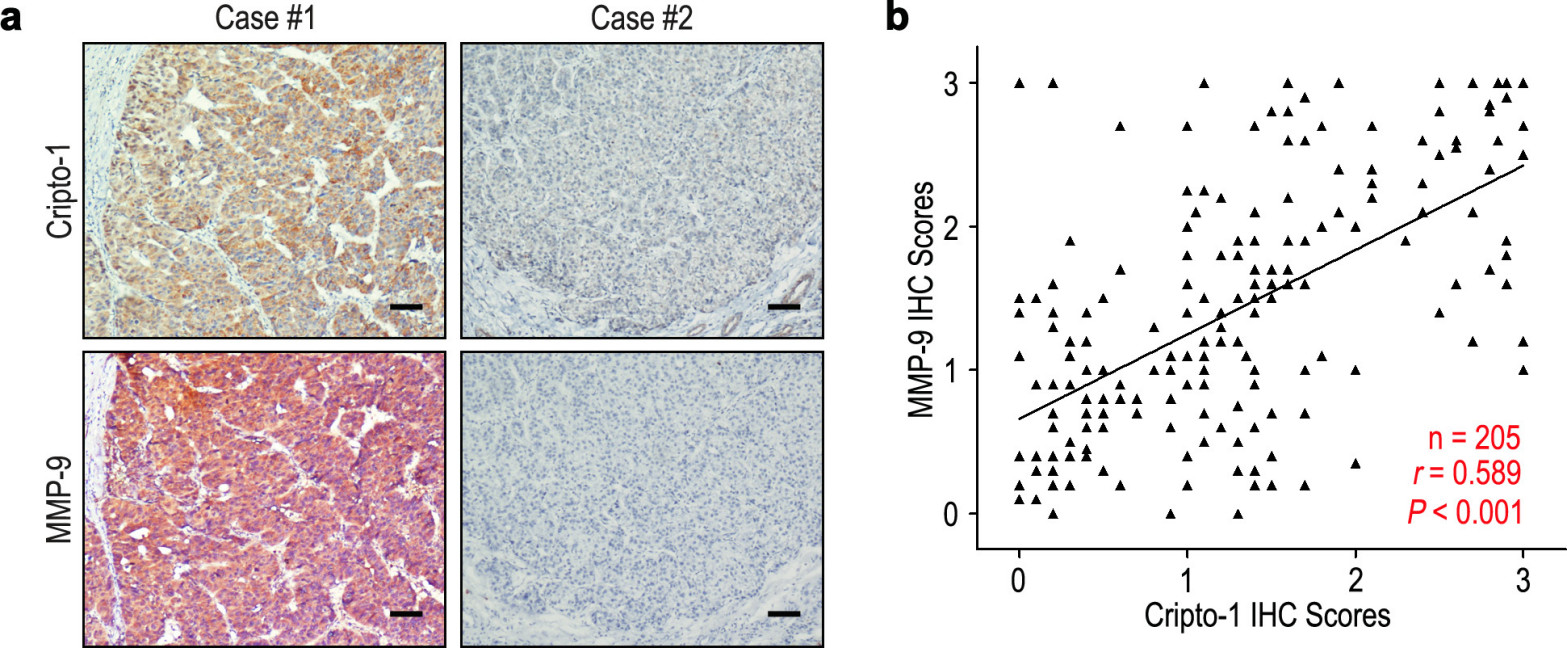
Cripto-1 and MMP-9 levels correlated in 205 HCC tissues **a.** Serial sections of human HCC tissue were subjected to IHC staining with antibodies against Cripto-1 and MMP-9. In case #1, high expression of Cripto-1 in HCC tissues was accompanied by elevated MMP-9. In case #2, low expression of Cripto-1 was accompanied by the absence of MMP-9. The scale bar represents 50 μm. **b.** Spearman correlation analysis between Cripto-1 and MMP-9 expression in 205 HCC patients by IHC assays. Cripto-1 expression was positively correlated with MMP-9 expression.

In addition, seventy-nine of 109 (72.5%) patients with low MMP-9 expression had low Cripto-1 expression, while 72 of 96 patients (75.0%) with high MMP-9 expression also had high Cripto-1 expression (*P* < 0.001, Table 1). Moreover, the relationship of Cripto-1 and MMP-9 was further confirmed by IHC assays in serial sections of HCC tissues (Figure 3a). The results showed that Cripto-1 was positively correlated with MMP-9 in 205 HCC samples (*r* = 0.589, *P* < 0.001, Figure 3b).

**Figure 4:**
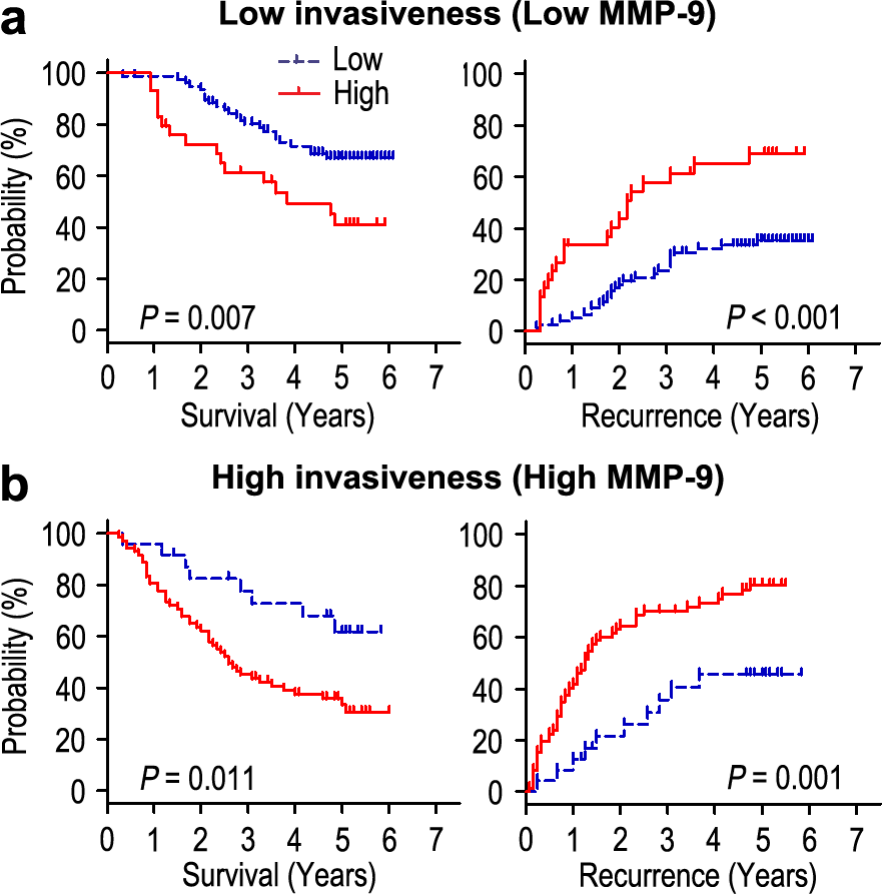
Overall survival and time to recurrence are shown for patients with low tumor aggressiveness **a.** and high tumor aggressiveness **b.** Kaplan-Meier survival estimates and log-rank tests were used to analyze the association between Cripto-1 expression and overall survival or time to recurrence in patients with low aggressiveness (low MMP-9; *n* = 109) or high aggressiveness (high MMP-9; *n* = 96).

We further investigated the impact of tumor aggressiveness on the prognosis of Cripto-1 expression in HCC by using MMP-9 marker as an indicator for invasive potential of tumor cells. The HCC patients were classified into either low aggressiveness group (low MMP-9 expression; *n* = 109) or high aggressiveness group (high MMP-9 expression; *n* = 96) based on the MMP-9 expression index. Kaplan-Meier survival curves were then plotted to determine the correlation of Cripto-1 expression and survival (Figure 4). In the low aggressiveness group, overexpression of Cripto-1 was correlated with poor OS (*P* = 0.007) and shorter TTR (*P* < 0.001) compared with the survival in low Cripto-1 expression patients (Figure 4a). In the high tumor aggressiveness group (Figure 4b), patients with high Cripto-1 expression were prone to death (*P* = 0.011) and relapse (*P* = 0.001). Therefore, the expression of Cripto-1 appears to be a strong postoperative prognostic parameter for patients with HCC independent of tumor aggressiveness.

### Prognostic significance of Cripto-1 on HCC early and late recurrence

Using 36 months postresection as the cut-off time, HCC recurrence was divided into an early recurrence that likely experienced intrahepatic metastasis due to dissemination of the primary tumor cells, and a late recurrence that was more likely to have de novo hepatocarcinogenesis [17]. The expression of Cripto-1 was higher in the early and late recurrence groups, compared with that in the no recurrence groups (*P* = 0.001 and *P* = 0.021, respectively) (Supplementary Figure S2). Univariate analysis showed that overexpression of Cripto-1 was significantly correlated with HCC early and late recurrence (both *P* < 0.001). Multivariate analysis confirmed that Cripto-1 had predictive validity for early recurrence (HR = 2.309, 95% CI = 1.309–4.073, *P* = 0.004) and late recurrence (HR = 2.839, 95% CI = 1.490–5.410, *P* = 0.002) (Table 3).

**Table 3:** Univariate and multivariate analysis of Cripto-1 expression associated with HCC early and late recurrence

Variables	Early recurrence				Late recurrence			
	Univariate	Multivariate			Univariate	Multivariate		
	*P*-value	*P*-value	HR	95% CI	*P*-value	*P*-value	HR	95% CI
Gender (Female vs. Male)	0.028	NS			NS	NS		
Age, years (≤50 vs. >50)	NS	NS			NS	NS		
AFP (ng/mL) (≤400 vs. >400)	0.003	0.016	1.885	1.125–3.158	NS	NS		
HBsAg (Negative vs. Positive)	0.028	0.023	4.199	1.215–14.514	NS	NS		
GGT (U/l) (≤50 vs. >50)	NS	NS			NS	NS		
Liver cirrhosis (No vs. Yes)	NS	NS			NS	NS		
Tumor size (cm) (≤5 vs. >5)	0.003	NS			0.044	NS		
Tumor number (Single vs. Multiple)	NS	NS			NS	NS		
Satellite nodule (No vs. Yes)	NS	NS			NS	NS		
Tumor capsule (No/incomplete vs. Complete)	NS	NS			NS	NS		
Tumor differentiation (I–II vs. III–IV)	NS	NS			NS	NS		
Vascular invasion (No vs. Yes)	<0.001	0.001	2.849	1.573–5.158	NS	NS		
Cripto-1 (Low *versus* High)	<0.001	0.004	2.309	1.309–4.073	<0.001	0.002	2.839	1.490–5.410

GGT gamma-glutamyltransferase, AFP α-fetoprotein, OS overall survival, TTR time to recurrence, NS not significant, HR hazard ratio, CI confidential interval.

### Combined influence of Cripto-1 and serum AFP on risk of HCC death and recurrence

**Figure 5:**
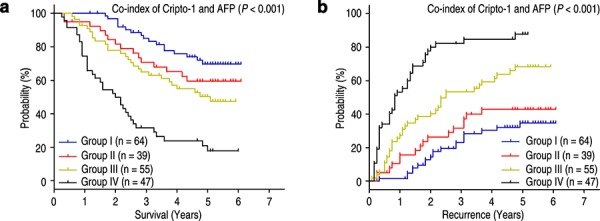
Combined influence of Cripto-1 and serum AFP on risk of HCC death and recurrence The associations of Cripto-1/AFP co-expression with Overall survival (log-rank *P* < 0.001) **a.** and Time to recurrence (log-rank *P* < 0.001) **b.** in 205 HCC patients.

It has been known that serum AFP levels are an unfavorable prognostic factor for HCC patients [16]. Univariate analysis indicated that preoperative serum AFP level above 400 ng/mL was significantly associated with shorter OS (*P* < 0.001) and TTR (*P* = 0.001) (Table 2). Therefore, we evaluated the prognostic value of Cripto-1 expression with serum AFP levels for recurrence and survival of HCC patients. Based on Cripto-1 expression and serum AFP values, HCC patients were categorized into four groups with different recurrent risks and prognosis: group I with Cripto-1 (−) and AFP ≤ 400 ng/mL, good prognosis and low-risk of recurrence; group II with Cripto-1 (−) and AFP > 400 ng/mL, and group III with Cripto-1 (+) and AFP ≤ 400 ng/mL, intermediate prognosis and intermediate-risk of recurrence; group IV, Cripto-1 (+) and AFP > 400 ng/mL, poor prognosis and high-risk of recurrence (Figure 5). Multivariate analysis further demonstrated that the combination of Cripto-1/AFP was an independent prognostic factor for OS (HR = 1.621, 95% CI = 1.327–1.980, *P* < 0.001) and TTR (HR = 1.638, 95% CI = 1.337–2.006, *P* < 0.001) (Table 4).

**Table 4:** Univariate and multivariate analysis of co-index of Cripto-1/AFP associated with survival and recurrence in HCC patients

Variables	OS				TTR			
	Univariate	Multivariate			Univariate	Multivariate		
	*P*-value	*P*-value	HR	95% CI	*P*-value	*P*-value	HR	95% CI
Gender (Female vs. Male)	NS	NS			NS	NS		
Age, years (≤50 vs. >50)	NS	NS			NS	NS		
HBsAg (Negative vs. Positive)	NS	NS			NS	NS		
GGT (U/l) (≤50 vs. >50)	0.009	NS			0.016	NS		
Liver cirrhosis (No vs. Yes)	0.006	0.021	2.060	1.116–3.803	0.012	0.018	2.104	1.138–3.890
Tumor size (cm) (≤5 vs. >5)	<0.001	NS			<0.001	0.048	1.617	1.004–2.604
Tumor number (Single vs. Multiple)	NS	NS			NS	NS		
Satellite nodule (No vs. Yes)	NS	NS			0.007	NS		
Tumor capsule (No/incomplete vs. Complete)	NS	NS			NS	NS		
Tumor differentiation (I–II vs. III–IV)	NS	NS			NS	NS		
Vascular invasion (No vs. Yes)	<0.001	<0.001	5.133	2.847–9.252	<0.001	<0.001	5.297	2.932–9.572
Co-index of Cripto-1/AFP*	<0.001	<0.001	1.621	1.327–1.980	<0.001	<0.001	1.638	1.337–2.006

* Co-index of Cripto-1/AFP was combined with Cripto-1 and AFP, therefore, we did not enter the Cripto-1 and AFP into univariate and multiple analysis with these indexes to avoid any bias in analysis.

GGT gamma-glutamyltransferase, AFP α-fetoprotein, OS overall survival, TTR time to recurrence, NS not significant, HR hazard ratio, CI confidential interval.

To enhance the diagnostic value of Cripto-1/AFP expression for early recurrence of HCC, we further performed a receiver-operating characteristic (ROC) curve analysis to determine the efficacy of Cripto-1 and serum AFP expression in discriminating early recurrence in HCC patients. The results showed that the area under the curve (AUC) was 0.602 for serum AFP and 0.735 for Cripto-1, while there was 0.751 for the Cripto-1/AFP combination (Supplementary Figure S3). In addition, we also compared the AUC of Cripto-1/AFP combination with serum AFP or Cripto-1, and found that the AUC for Cripto-1/AFP combination was significantly bigger than that of serum AFP, while there was no difference when compared with the AUC of Cripto-1 (Data not show). Our data suggested that the AUC for the combined marker may be superior to serum AFP alone.

## DISCUSSION

Human Cripto-1 regulates essential steps in early embryogenesis, and promotes cell migration, angiogenesis, and stem cell maintenance [7, 18]. Expression of Cripto-1 mRNA and/or protein has been found in human tumors including colorectal [13], breast [19], gastric [14], bladder [20] and lung carcinomas [21]. Interestingly, Numerous studies have demonstrated high expression levels of Cripto-1 to correlate with malignant transformation, tumor invasiveness, metastatic spreading, and poor prognosis [14, 22, 23]. In addition, it has been reported that Cripto-1 is involved in regulation of EMT and invasion ability in HCC cells [24]. However, the prognostic significance of Cripto-1 expression in HCC is still unclear. In current study, we found that Cripto-1 was up-regulated in HCC tissues compared with adjacent non-tumorous tissues. In addition, Cripto-1 overexpression was significantly associated with larger tumor, TNM stage, BCLC stage and tumor recurrence. Moreover, the Kaplan-Meier survival analysis showed that the OS and TTR of HCC patients with high Cripto-1 expression were shorter than those with low Cripto-1 expression. The expression levels of Cripto-1 were significantly increased in TNM and BCLC stage-dependent manner. According to the results of multivariate analysis, we found that Cripto-1 overexpression was an independent predictor for poor OS as well as TTR in HCC patients. Furthermore, the prognostic value of Cripto-1 in different subgroups based on tumor size, tumor differentiation, TNM stage and BCLC stage was also estimated, which appeared that Cripto-1 may serve as a powerful prognostic factor for patients with HCC in different risk groups. The results indicated that Cripto-1 could serve as a feasible prognostic biomarker of HCC. Our findings had similar results with previous study. Sun C et al. [24] reported that Cripto-1 knockdown resulted in increased expression of E-CADHERIN and decreased expression of P-SMAD3, N-CADHERIN, VIMENTIN and SNAIL in Huh7 and SMMC7721 cells. Overexpression of Cripto-1 led to decreased expression of E-CADHERIN and increased expression of P-SMAD3, N-CADHERIN, VIMENTIN and SNAIL in Huh7 and SMMC7721 cells. Moreover, Cripto-1 knockdown also repressed the invasion ability of Huh7 and SMMC7721 cells, while overexpression of Cripto-1 enhanced the invasion ability of Huh7 and SMMC7721 cells. Our findings and previous observations strongly implicate that Cripto-1 overexpression is involved in the tumor progression and may serve as a prognostic factor for HCC patients.

MMP-9 was shown to boost the bioavailability of growth factors and to disrupt cell-cell contacts, dramatically affecting cell proliferation and survival [25]. Arii S et al [15]. reported that the expression of MMP-9 mRNA in HCC with capsular infiltration was significantly higher than in HCC without capsular infiltration. In addition, MMP-9 immunoreactivity was the most intense in the HCC cells, particularly in those cells in the marginal areas of the tumorous tissues. MMP-9 is an independent predictor of tumor recurrence and survival in HCC patients [26]. Our results showed that Cripto-1 was positively associated with MMP-9 protein expression in HCC tissues. Moreover, the tumor cells with Cripto-1 overexpression revealed high aggressiveness, and high Cripto-1 expression was a strong predictor of poor prognosis in patients with HCC independent of tumor cell aggressiveness (Figure 4). Collectively, Cripto-1 status in HCC promoting tumor progression indicates that Cripto-1 can be a potential target in cancer therapy.

Intrahepatic recurrence of HCC after hepatectomy could originate from either intrahepatic metastasis (IM) from the primary tumor or multicentric occurrence (MO) [27, 28]. According to the different time after surgery, intrahepatic recurrence can be classified into early and late type. Previous studies have shown that early recurrence might represent mainly IM, whereas late recurrence represented mainly MO [4]. Furthermore, early recurrence was likely to correlate with aggressive tumor biology, especially in vascular infiltration [29, 30], while late recurrence was likely to associate with the presence of cirrhosis [30] and hepatitis virus infection [31]. However, molecular markers for predicting early and late recurrence are still unknown. In our study, by comparative analysis of expression profiles with or without tumor recurrence, Cripto-1 was found to be concordantly upregulated in early and late recurrence HCC, and could be an independent biomarker for predicting early and late recurrence of HCC patients. In general, Cripto-1 expression had predictive validity for early and late recurrence in HCC.

AFP is a useful tumor-associated antigen for the diagnosis and predicted prognosis of HCC and monitoring metastasis and tumor recurrence in HCC patients with high AFP after hepatectomy [32, 33]. However, it is difficult to predict the prognosis and metastatic recurrence of normal AFP HCC patients after hepatectomy. To investigate whether the prognostic value of Cripto-1 combined with serum AFP level was superior to AFP alone, we divided the HCC patients into four groups according to Cripto-1 expression and serum AFP level and found that combination of Cripto-1 and serum AFP level could be used for predicting the risk of tumor recurrence and survival of patients. HCC patients can be classified to different subgroups with different risks of tumor recurrence and prognosis according to Cripto-1 expression in HCC tissue and preoperative AFP level. Moreover, our analysis of the Cripto-1 /AFP combination as a predictor for early recurrence showed that the sensitivity and specificity may be superior to AFP alone. The simultaneous analysis of Cripto-1 expression and serum AFP level might help determine whether adjuvant therapy is required after resection.

In conclusion, our results revealed that Cripto-1 may have a pivotal role in tumor aggression and prognosis, and can act as a feasible biomarker for prognostic prediction in HCC. Moreover, the combination of Cripto-1 with serum AFP level may help to identify the high-risk HCC patients after hepatectomy. The findings of the current study may help to determine optimal treatment strategies. However, it also requires further studies to clarify the underlying biology of Cripto-1 in the development of HCC.

## MATERIALS AND METHODS

### Patients and specimens

The study was approved by the Institutional Review Board and Human Ethics Committee of Sun Yat-Sen University Cancer Center. Written consent for using the samples for research purposes was obtained from all patients prior to surgery.

All HCC tissues and their adjacent non-tumorous liver tissues were collected from 205 consecutive patients who had received curative liver resection from January 2008 to December 2008 at the Department of Hepatobiliary Oncology, Sun Yat-sen University (Guangzhou, China). The eligibility criteria of the current study were as follows: (1) all the tumor and adjacent non-tumorous tissues were confirmed histologically, (2) none of the patients had distant metastasis or received previous radiotherapy and chemotherapy before hepatectomy, (3) no serious complications or other malignant diseases. The cases were selected consecutively on the basis of availability of resection tissues and follow-up data. Detailed clinicopathological features were obtained from patients’ files (Supplementary Table S1). Tumor stage was classified according to the 7th Edition tumor-node-metastasis (TNM) classification of the American Joint Committee on Cancer Staging and the Barcelona Clinic Liver Cancer (BCLC) staging system. Overall survival (OS) was computed from the date of surgery to the date of death or last follow-up. Time to recurrence (TTR) was defined as from the date of surgery to the date of relapse, metastasis, or last follow-up.

### IHC staining

A total of 205 HCC tissues and their adjacent non-tumorous samples were used in the IHC analysis. Formalin-fixed, paraffin-embedded specimens from consenting patients were cut in 4 μm sections. After being baked at 60°C for 2 h, the samples were deparaffinized in xylene and rehydrated using a series of graded alcohols. Then, the tissue slides were incubated with 3% hydrogen peroxide for 10 min to exhaust endogenous peroxidase activity. The sections were microwaved for antigen retrieval in 0.01 M sodium citrate buffer (pH 6.0) for 30 min, and then preincubated in 10% normal goat serum for 30 min to prevent nonspecific staining. The sections were incubated with the Cripto-1 rabbit polyclonal antibody (working dilution 1:100, Abcam, #ab19917, UK) and MMP-9 rabbit polyclonal antibody (working dilution 1:200, Abcam, #ab38898, UK) overnight at 4°C. Subsequently, the sections were treated with a biotinylated goat anti-rabbit secondary antibody (DAKO, Glostrup, Denmark) for 30 minutes at 37°C. Assessments of the staining were scores by two experienced pathologists blinded to the patients’ identity and clinical status. In discrepant cases, a pathologist reviewed the cases and reached the consensus.

Both the extent and intensity of immunostaining were taken into consideration when analyzing the data. The intensity of staining was scored from 0 to 3, and the extent of staining was scored from 0% to 100%. The final quantitation of each staining was obtained by multiplying the two scores. Cripto-1 expression was classified as high expression if the score was higher than the median score of 1.2, if the score was 1.2 or less, the case was classified as low expression. MMP-9 expression was considered high if the score was higher than 1.4.

### Follow-up

The last follow-up was on 30 February 2014. In all the HCC patients (24 females and 181 males), the median follow-up period was 46.0 months, ranging from 3 to 73 months. All patients were followed up every 1–3 months in the first year and every 3–6 months thereafter. The follow-up protocol included physical examination, serum alpha-fetoprotein (AFP) level, chest X-ray, and abdominal ultrasonography. Computed tomography and/or magnetic resonance imaging and/or positron emission tomography were performed when intrahepatic relapse or distant metastasis was suspected. The main causes of death were HCC recurrence or complicated cirrhosis of the liver. During the course of follow-up, 112 of 205 HCC patients (54.6%) were found with recurrence and 95 patients (46.3%) died of cancer-related causes. One hundred and ten patients were still alive at the time of the last follow-up report.

### Statistical analysis

The SPSS software package (version 16.0; Chicago, IL, USA) was used for the statistical analysis. The chi-square test was used to analyze the correlation of Cripto-1 status with clinicopathological features. The Student's *t*-test was used for comparisons. Pearson χ2 test was applied to analyze the correlation of Cripto-1 with MMP-9 staining scores. Survival curves were generated using the Kaplan-Meier method, and differences between curves were estimated by the log-rank test. The Cox multivariate proportional hazards regression model was used to determine the independent factors that influence survival and recurrence based on the investigated variables. ROC curve analysis was used to determine the efficacy of Cripto-1 and serum AFP expression in discriminating early recurrence (less than 3 years) from HCC patients. All *P* values were two-sided and *P* values less than 0.05 was considered to be statistically significant.

## SUPPLEMENTARY FIGURES AND TABLE


