# Supplementary material for: Elevated expression of Cripto-1 correlates with poor prognosis in hepatocellular carcinoma
Source: Oncotarget. 2015 Sep 5;6(33):35116–28. doi: 10.18632/oncotarget.5057 (PMC4741514; doi:10.18632/oncotarget.5057)
Supplement: Supplementary file 1 [file oncotarget-06-35116-s001.pdf]

SUPPLEMENTARY FIGURES AND TABLE

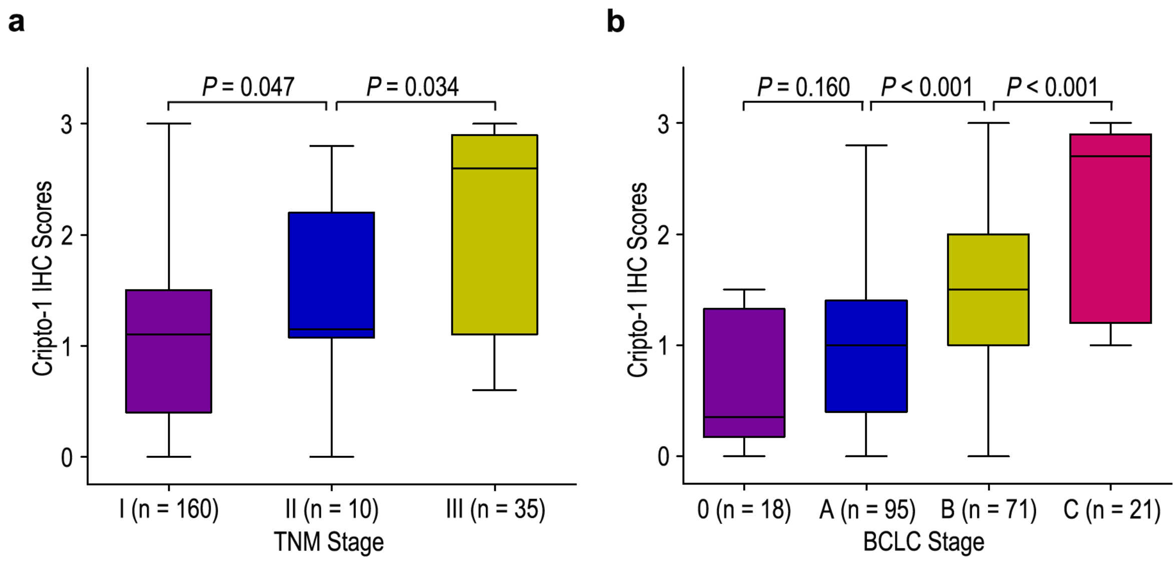

Supplementary Figure S1: Comparsion of Cripto-1 expression by TNM stage and BCLC stage. Cripto-1 expression is markedly increased as tumors progress in TNM stage **a.** and BCLC stage **b.** Statistical analysis was performed by Student's *t*-test.

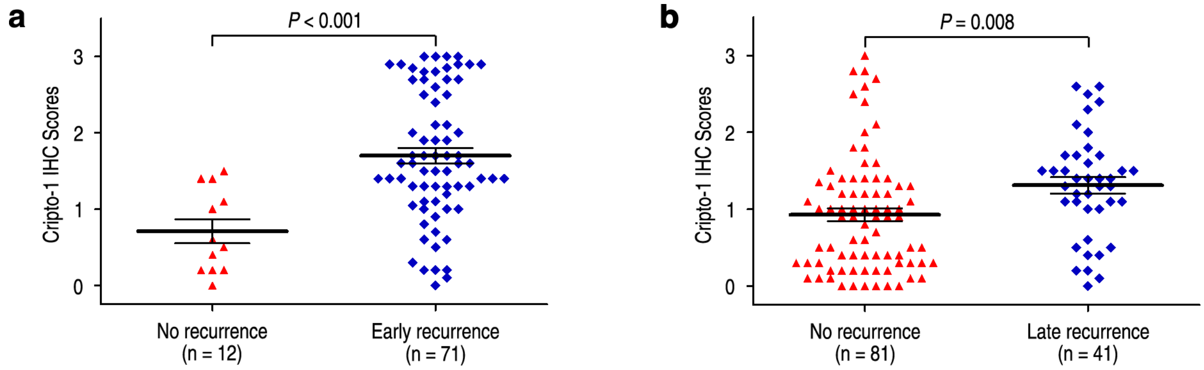

Supplementary Figure S2: Comparsion of Cripto-1 expression in HCC patients with or without recurrence. The expression of Cripto-1 is higher in early recurrence **a.** and late recurrence **b.** patients. Statistical analysis was performed by Student's *t*-test.

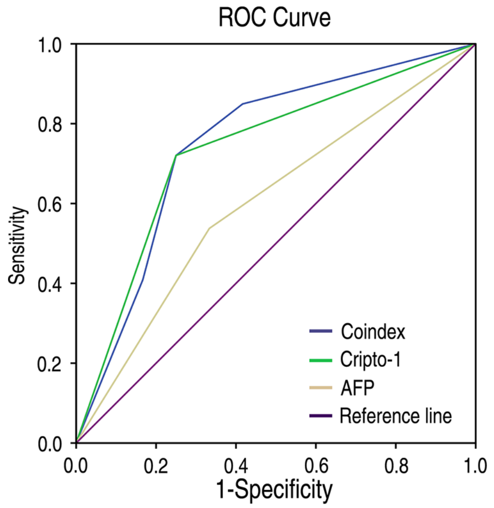

Supplementary Figure S3: ROC curve analysis of individual markers and combination of Cripto-1 and serum AFP for discriminating early recurrence from HCC patients. AUC was 0.602 for serum AFP ($P = 0.251$), 0.735 for Cripto-1 ($P = 0.008$), 0.751 for Cripto-1 and serum AFP combinations ($P = 0.005$).

Supplementary Table S1: Patient Characteristics

Variable	No. of patients (%)
No. of patients	205 (100)
Age: Median [range], y	47 [13–78]
**Gender**	
Female	24 (11.7)
Male	181 (88.3)
**HBsAg**	
Negative	13 (6.3)
Positive	192 (93.7)
AFP: Median [range], ng/mL	159.2 [0.6–121000]
GGT: Median [range], U/l	45.0 [3.5–655.5]
Tumor size: Median [range], cm	4.5 [1.0–18.0]
**Liver cirrhosis**	
No	45 (22.0)
Yes	160 (78.0)
**Child-Pugh class**	
A	200 (97.6)
B	5 (2.4)
**Tumor number**	
Single	189 (92.2)
Multiple	16 (7.8)
**Satellite nodule**	
No	178 (86.8)
Yes	27 (13.2)
**Tumor capsule**	
No/incomplete	121 (59.0)
Complete	84 (41.0)
**Tumor differentiation**	
I	11 (5.4)
II	131 (63.9)
III	56 (27.3)
IV	7 (3.4)
**Vascular invasion**	
No	185 (90.2)
Yes	20 (9.8)

(*Continued*)

Variable	No. of patients (%)
**TNM stage**	
I	160 (78.0)
II	10 (4.9)
III	35 (17.1)
**BCLC stage**	
0	18 (8.8)
A	95 (46.3)
B	71 (34.6)
C	21 (10.2)
**Recurrence status**	
No	93 (45.4)
Early recurrence	78 (38.0)
Late recurrence	34 (16.6)
Late recurrence	34 (16.6)
